# BRAF p.Val600Glu (V600E) Testing for Assessment of Treatment Options in Metastatic Colorectal Cancer

**DOI:** 10.1371/currents.RRN1187

**Published:** 2010-11-26

**Authors:** Andrew Lea, Diane Allingham-Hawkins, Susan Levine

**Affiliations:** Hayes, Inc.

## Abstract

Colon and rectal cancer (CRC) are the third most common cancer in the United States and cause approximately 50,000 deaths per year. The anti–epidermal growth factor receptor (EGFR) monoclonal antibodies cetuximab (Erbitux®) and panitumumab (Vectibix®) have been recently introduced to treat CRC. However, the response rate with these agents is low and they are associated with serious adverse effects. Accordingly biomarkers that can predict those patients that will respond to treatment may have clinical utility. The p.Val600Glu sequence variant (often called V600E) in the BRAF gene has been investigated as a biomarker to predict patients that will not respond to treatment with the anti-EGFR monoclonal antibodies.

## **Clinical Scenario**

Together, colon and rectal cancer (colorectal cancer, or CRC) comprise the third most common cancer in the United States, with an estimated 50,000 deaths caused by CRC in 2009[1] [2] [3] [4]. The 5-year survival rate for the 150,000 individuals diagnosed each year with CRC is 65.2% for all stages, but drops to 11.3% in those with metastatic disease[2]. Single or multiagent chemotherapy regimens may be chosen based on the drugs that are currently approved for treating metastatic CRC, which include bevacizumab, capecitabine, cetuximab, fluorouracil, irinotecan, oxaliplatin, and panitumumab[3] [4] [5] [6]. Cetuximab (Erbitux®; Imclone Systems Inc./Bristol-Myers Squibb) and panitumumab (Vectibix®; Amgen Inc.) are anti–epidermal growth factor receptor (EGFR) monoclonal antibodies that may be used for first-, second- or third-line treatment in patients with metastatic disease[7] [8] [9]. EGFR is a component of the HER/Erb-B (human EGFR) signaling pathway[10]. Clinical evidence suggests that benefit from the EGFR monoclonal antibody inhibitors cetuximab and panitumumab is limited to a subgroup of only 10% to 30% of CRC patients[11] [12] [13]. Biomarkers are therefore needed to help select the patients who will benefit from treatment with EGFR inhibitors[9]. In July 2009, the presence of sequence variants in the Kirsten rat sarcoma viral oncogene homolog (*KRAS*) gene has been approved by the Food and Drug Administration (FDA) as a negative prognostic indicator for response to treatment with cetuximab and panitumumab[13]. Another biomarker that is part of the same EGFR signaling pathway as *KRAS *that has been investigated for predicting response to EGFR monoclonal antibody treatment in CRC is the v-raf murine sarcoma viral oncogene homolog B, known as *BRAF* [14] [15].The *BRAF *gene sequence variant p.Val600Glu, often called V600E, is the main sequence variant in this gene that is associated with CRC[16]. The frequency of the *BRAF *p.Val600Glu variant was 5% to 12% in CRC populations, and the presence of this variant has been found to be associated with poor survival in patients with CRC in several studies in which patients were not treated with anti-EGFR monoclonal antibodies[16] [17] [18] [19] [20] [21] [22] [23]. Furthermore, there appears to be little variation in the *BRAF *p.Val600Glu status between primary and secondary tumors[23] [24]. As testing for the presence of *KRAS *sequence variants to predict response to therapy with the anti-EGFR monoclonal antibodies cetuximab and panitumumab is now included in the FDA prescribing information, this Knol focuses on genetic testing for *BRAF *p.Val600Glu following *KRAS *testing.

##  **Test Description**

A number of providers have assays that can detect the *BRAF* p.Val600Glu sequence variant from formalin-fixed paraffin-embedded (FFPE) tumor samples or slides using a variety of technologies, including polymerase chain reaction (PCR) with and without fluorescence monitoring, dideoxy sequencing, direct sequencing, and pyrosequencing:

ARUP Laboratories (Salt Lake City, UT) *BRAF *V600E Mutation Detection by PCR (2002498): This assay requires FFPE tumor tissue block or at least 3 unstained 5-micrometer (μm) slides of tumor tissue, and uses PCR with pyrosequencing to detect sequence variants in the *BRAF *gene[25]. If *KRAS *sequence variants are not detected in a specimen, this assay can also be requested by selecting the *KRAS *Mutation Detection with *BRAF *reflex (2001932) test[26].

Laboratory Corporation of America (LabCorp) (Burlington, NC) *BRAF *Gene Mutation Detection Test (480340): This assay requires either an FFPE tissue block with ≥ 50% tumor or 4 unstained 5-μm slides and a single hematoxylin and eosin (H&E)-stained 5-μm slide. The assay uses dideoxy sequencing to detect the *BRAF *p.Val600Glu sequence variant[27]. If *KRAS *sequence variants are not detected in a specimen, this assay can also be requested by selecting the K-ras Gene Mutation Detection With Reflex to *BRAF *Gene Mutation Detection Test (480360)[27].

Mayo Medical Laboratories (Rochester, MN) *BRAF *Mutation Analysis (V600E), Tumor (Unit Code 87980): The specimen requirements for this assay are an FFPE tumor tissue block or a single H&E-stained 5-μm slide with 10 unstained nonbaked slides with 5 slides, each with 5-μm and 10-μm-thick sections. The assay uses PCR to detect the *BRAF *p.Val600Glu sequence variant[28].

- Quest Diagnostics Inc. (Madison, NJ) *BRAF *Mutation Analysis (16767): This assay requires an FFPE tumor tissue block. Direct sequencing is used to detect sequence variants in the *BRAF *gene[29]. This assay can also be requested by selecting the EGFR Pathway (*KRAS *with Reflex to *NRAS*, *BRAF*) (16819) assay if the specimen is found to be *KRAS *sequence variant-negative[30].
- UNC Health Care McLendon Clinical Laboratories (Chapel Hill, NC) *BRAF *Mutation Test in Colorectal Cancer: The specimen preferred for this assay is an FFPE tumor tissue block containing ≥ 50% malignant cells or 10 5- to 10-μm slides with a single H&E-stained slide. The assay uses PCR followed by pyrosequencing to detect the *BRAF *p.Val600Glu sequence variant[31].

Vanderbilt Pathology Laboratory Services (Nashville, TN) *BRAF* (V600E) (V6A): The assay requires an FFPE tissue block and uses allele-specific detection to identify the *BRAF* p.Val600Glu sequence variant. Also available is a second assay, the *BRAF* (V600E Sequencing) (V6S), which uses sequencing to detect the *BRAF *p.Val600Glu sequence variant[32].

## **Public Health Importance**

Available evidence indicates that the clinical benefit from treatment with EGFR monoclonal antibody inhibitors cetuximab and panitumumab is limited to a subgroup of only 10% to 30% of CRC patients[11] [12] [13]. Accordingly, biomarkers are needed to help select patients who will benefit from treatment with EGFR monoclonal antibody inhibitors and also to avoid unnecessary exposure of patients to the serious adverse events associated with these agents[9].

## **Published Reviews, Recommendations and Guidelines**

**Systematic evidence reviews**

None identified.

**Recommendations by independent group**

None identified.

**Guidelines by professional groups**

If patients do not have a sequence variant in the *KRAS *gene, the National Comprehensive Cancer Network (NCCN) guidelines for patients undergoing treatment for CRC state that testing for *BRAF *p.Val600Glu should be considered prior to the use of anti-EGFR antibodies (cetuximab and panitumumab). Furthermore, these guidelines indicate that patients with a *BRAF *sequence variant are unlikely to respond to treatment with anti-EGFR antibodies; however, it is noted that the data are somewhat inconsistent[5] [6].

## **Search Strategy**

Evidence evaluated for this report was obtained primarily from a search of the peer-reviewed literature in MEDLINE and EMBASE databases on September 18, 2010, using the terms (*V600E* OR *BRAF*) AND (*colon* OR *colorectal* OR *rectal*) AND *cancer*. Limits used were English language and published since January 1, 1996.

## **Evidence Overview**

***Analytic Validity*: **Test accuracy and reliability in measuring the presence or absence of the *BRAF* p.Val600Glu sequence variant (analytic sensitivity and specificity).

The analytic sensitivity and specificity of the commercially available assays are not reported on manufacturer websites[25] [26] [27] [28] [29] [30] [31] [32]. Several recent reports are available that describe studies of the analytical validity of genetic testing for *BRAF* sequence variants[33] [34] [35] [36] [37] [38]. However, these reports appear to have little bearing on the commercially available assays for the *BRAF *p.Val600Glu sequence variant. Two reports describe studies investigating testing for multiple biomarkers, including sequence variants in the *BRAF *gene, and, therefore, have no relevance to the commercially available assays that detect the *BRAF *p.Val600Glu sequence variant[34] [35]. Two other reports investigate the analytical accuracy of the technique of pyrosequencing[33] [36], a technology that is used only by UNC Health Care McLendon Clinical Laboratories. The report by Tan and colleagues (2008) suggests that pyrosequencing was able to detect the *BRAF* p.Val600Glu sequence variant at a tenfold lower variant-to-normal ratio than dideoxy sequencing, as well as being quicker and less costly to perform[33]. Packham and colleagues (2009) found that pyrosequencing had a sensitivity of 94.9% and a specificity of 100% in comparison to real-time PCR in detecting the *BRAF *p.Val600Glu sequence variant[36]. Jakubauskas and colleagues (2010) investigated the possibility of using multiplex PCR followed by dideoxy-termination sequencing to simultaneously detect *KRAS *sequence variants and the *BRAF *p.Val600Glu sequence variant[38], a technique not used in any of the commercially available assays. The final report compared the use of PCR followed by high-resolution melting (HRM) analysis, with the more traditional techniques of PCR followed by denaturing high-performance liquid chromatography (dHPLC), conventional PCR followed by direct sequencing, and real-time allele-specific PCR (RT-PCR)[37]. Although the complete results of this study were not clearly presented, the authors concluded that, for the detection of *BRAF* p.Val600Glu sequence variants, HRM was more sensitive than both DNA sequencing and dHPLC, and similar to RT-PCR[37].

***Clinical Validity*: **Test accuracy and reliability in identifying patients that will respond to treatment with EGFR monoclonal antibody inhibitors (predictive value).

Published studies of the clinical validity of *BRAF *p.Val600Glu testing were retrospective analyses of patients treated with cetuximab or panitumumab at cancer treatment centers[19] [39] [40] [41] [42] [43] or a cohort of patients that had been in one of three clinical trials[44](see Table 1). Response to treatment was generally defined using the Response Evaluation Criteria in Solid Tumors (RECIST) system, which classifies patients as having complete response (CR), partial response (PR), stable disease (SD), or progressive disease (PD)[45]. Patients are defined as having responded to treatment if they have either CR or PR. Other endpoints commonly used were progression-free survival and overall survival.

**Table 1. Overview of studies that examined the clinical validity of testing for *BRAF *p.Val600Glu for assessment of treatment options for colorectal cancer (CRC). Response to treatment was measured using the RECIST criteria[45].**

**Key:** CI, confidence interval; FOLFIRI, folinic acid, fluorouracil, and irinotecan; IR, irinotecan; OR, odds ratio; OS, overall survival; PCR, polymerase chain reaction; PFS, progression free survival; pt(s), patient(s).

**Table:** 

**Study**	**Protocol and Methods **	**Results for *KRAS *and *BRAF* analyzes **	**Comments**
De Roock and colleagues (2010)[46]	Retrospective analysis of pts treated with cetuximab in combination with chemotherapy at 11 European centers between 2001 and 2008. The primary endpoint was objective response, with PFS as a secondary endpoint. Sequence variants in the *KRAS *and *BRAF *genes were identified using multiple PCR with MALDI-TOF MassARRAY Type 4 Analyzer (Sequenom Inc., San Diego).	*KRAS and BRAF *sequence variants were found in 299 of 747 (40.0%) and 35 of 761 (4.7%) evaluable pts, respectively. *BRAF *sequence variants occurred only in pts without *KRAS *sequence variants. In pts without *KRAS *sequence variants: Response to treatment was lower in pts with *BRAF* sequence variants than without (8.3% versus 38.0%; OR 0.15; 95% CI, 0.02-0.51; P=0.0012) Median PFS was shorter in pts with *BRAF *sequence variants than without (8 wks versus 26 wks; HR, 3.74; 95% CI, 2.44-5.75; P<0.0001).	Approximately one third of pts included in this study had been included in previous reports. Of the 2 pts that responded to treatment with *BRAF *sequence variants, 1 had a p.Asp549Gly variant and the other had the p.Val600Glu variant, but at a low copy number in the tumor sample.
Laurent-Puig and colleagues (2009)[43]	Retrospective analysis of pts treated with cetuximab plus IR or FOLFIRI at 6 French hospitals. Primary or secondary endpoints were not defined. *KRAS *and *BRAF *sequence variants were identified using allelic discrimination with TaqMan probes.	*KRAS *sequence variants were found in 53 of 169 (31.4%) pts. *BRAF* sequence variants were identified in 5 of 115 (4.3%) evaluable *KRAS*-negative pts. In pts without *KRAS *sequence variants: No pts with *BRAF* sequence variants responded to treatment compared with 51 of 110 (46.4%) without *BRAF* sequence variants. PFS was shorter in pts with than without *BRAF* sequence variants (8.0 wks versus 31.4 wks; P<0.001). OS was also shorter in pts with than without *BRAF* sequence variants (6.5 mos versus 14.8 mos; P<0.001).	In this study, the presence of *KRAS* sequence variants was found to be a significant predictor of response to treatment, PFS and OS.
Loupakis and colleagues (2009)[39]	Retrospective analysis of pts treated at cancer treatment centers with cetuximab plus IR who had previously tested negative for *KRAS* sequence variants. Primary or secondary endpoints were not defined. PCR with pyrosequencing was used to identify *BRAF* sequence variants.	*BRAF *sequence variants were found in 13 of 87 (14.9%) pts without *KRAS* sequence variants. Response to treatment was lower in pts with than without *BRAF* sequence variants (0.0% versus 27.6%; P=0.016). Median PFS was shorter in pts with than without *BRAF* sequence variants, but not significantly (2.6 mos versus 4.4 mos; P=0.073). Median OS was significantly shorter in pts with than without *BRAF* sequence variants (4.1 mos versus 13.9 mos; HR 0.51; 95% CI, 0.18-0.95; P=0.037).	By the time of this analysis, 93% of pts had experienced disease progression and 72% had died.
Perrone and colleagues (2009)[42]	Retrospective analysis at a single Italian center of pts that were refractory to IR and treated with cetuximab plus IR. Primary or secondary endpoints were not defined. *BRAF *and *KRAS *sequence variants were identified using specific primers with PCR amplification.	*KRAS* sequence variants were identified in 7 of 29 (24.1%) of pts. *BRAF* sequence variants were found in 2 of 31 (6.4%) pts of which 1 pt responded to treatment and the other did not.	Across all pts, response to treatment with cetuximab was experienced by 10 of 32 (31.2%) pts.
Sartore-Bianchi and colleagues (2009)[40]	Retrospective analysis of pts treated with cetuximab or panitumumab at an Italian and a Swiss cancer center. Primary or secondary endpoints were not defined. PCR amplification with specific primers followed by automated sequencing by ABIPRISM 3730 (Applied Biosystems, Foster City, CA) was used to identify *KRAS* and *BRAF* sequence variants.	*KRAS* and *BRAF* sequence variants were identified in 35 of 132 (26.5%) and 11 of 132 (8.3%) of pts respectively. No pts had both *KRAS* and *BRAF* sequence variants. The relationship between *BRAF* sequence variants and response to treatment in pts without *KRAS* sequence variants was not significant (OR, 0.24; 95% CI, 0.00-3.09; P=0.265). PFS was not significantly different between those with and without *BRAF* sequence variants (P=0.22), however, OS was longer in pts without than with *BRAF* sequence variants (HR 3.75; 95% CI, 1.29-10.90; P=0.015).	109 pts received cetuximab, with 23 pts receiving panitumumab. This report came from the same research group as the report by Di Nicolantonio and colleagues[41] and may have included some of the same pts.
Souglakos and colleagues (2009)[19]	Retrospective analysis of pts treated at 2 cancer centers in the United States and Greece; 100 pts received a variety of chemotherapy regimen that included cetuximab. Primary or secondary endpoints were not defined. Sequence variants in *KRAS *and *BRAF *were identified using PCR followed by mass-spectrometric genotyping or Sanger sequencing.	*KRAS* sequence variants were found in 32 of 92 (34.8%) of pts. *BRAF* sequence variants were identified in 9 of 92 (9.8%) of pts. No pts that had *BRAF* sequence variants responded to treatment compared to 14 of 83 (16.9%) without *BRAF* sequence variants. PFS was significantly shorter in those with than without *BRAF* sequence variants that were treated with first-line cetuximab regimens (2.0 mos versus 3.9 mos; HR 3.6; 95% CI 1.8-7.4).	Of the 100 pts that received cetuximab, it was as first-line (n=8), second-line (n=37) or third-line or higher therapy (n=55).
Di Nicolantonio and colleagues (2008)[41]	Retrospective analysis of pts treated with cetuximab or panitumumab at an Italian and a Swiss cancer center. Primary or secondary endpoints were not defined. PCR amplification with specific primers followed by automated sequencing by ABI PRISM 3730 was used to identify *KRAS* and *BRAF* sequence variants.	*KRAS* sequence variants were found in 34 of 113 (30.1%) of pts. *BRAF* sequence variants were found in 11 of 79 (13.9%) evaluable pts. The presence of *BRAF* and *KRAS* sequence variants was mutually exclusive. Response to treatment was lower in pts with than without *BRAF* sequence variants (0.0% versus 32.4%; P=0.029). In pts without *KRAS *sequence variants, an absence of *BRAF* sequence variants was associated with increased PFS (P=0.010) and OS (P<0.0001) in Kaplan-Meier analyzes.	87 pts received cetuximab and 26 received panitumumab. Over all pts in the study, response to treatment was seen in 24 of 133 (21.2%) of pts and the median duration of response was 25 wks.
Freeman and colleagues (2008)[44]	Retrospective analysis of 62 of the 533 pts involved in phase II clinical trials that received panitumumab in the United States. Primary or secondary endpoints were not defined. *BRAF* and *KRAS* sequence variants were analyzed using PCR and sequencing.	*KRAS *sequence variants were identified in 24 of 62 (38.7%) pts. *BRAF* sequence variants were found in 4 of the 62 (6.4%) pts. The *KRAS* status of these pts was not clearly reported. Of the 4 pts with *BRAF* sequence variants, 1 had a partial response, 1 had stable disease and 2 had progressive disease.	Across all pts, response to treatment with panitumumab was observed by 4 of 62 (6.5%) pts.

A meta-analysis of 4 of the above studies (Di Nicolantonio[41], Laurent-Puig[43], Loupakis[39], and Sartore-Bianchi[40]) was conducted in 2010 by Mao and colleagues to examine the relationship between *BRAF *sequence variants and response to treatment with the EGFR monoclonal antibody inhibitors[47]. The results of the meta-analysis indicate that in patients without *KRAS *sequence variants, the objective response rate in patients with the *BRAF *p.Val600Glu sequence variant was 0.0% (0 of 40); it was 36.3% (122 of 336) in patients without *BRAF* sequence variants. This resulted in a pooled risk ratio of 0.14 (95% CI,  0.04 to 0.53; P=0.004). For progression-free survival, the difference between those with and without *BRAF* sequence variants was not significant across the 2 studies that reported this endpoint. However, overall survival was shorter for those patients with *BRAF *sequence variants compared to those without (HR, 3.25; 95% CI, 1.63 to 6.47; P=0.001)[47].

***Clinical Utility*: **Net benefit of test in improving health outcomes.

No reports that prospectively investigated the clinical utility of genetic testing for the *BRAF *p.Val600Glu sequence variant were identified. If detection of the *BRAF *p.Val600Glu sequence variant can be used to predict a lack of response to treatment using cetuximab and panitumumab, such testing would have clinical utility in avoiding exposure to the EGFR monoclonal antibody inhibitors, which are associated with serious adverse effects, in patients who are unlikely to benefit. A potential harm of testing for the *BRAF *p.Val600Glu sequence variant is if the presence of this variant does not accurately predict a lack of response to the anti-EGFR monoclonal antibody, a potentially efficacious treatment would be denied to *BRAF *p.Val600Glu-positive patients.

## **Conclusions**

- Data that define the analytical validity of the commercially available assays for the *BRAF *p.Val600Glu sequence variant are limited.
- The clinical validity of testing for the *BRAF *p.Val600Glu sequence variant to predict response to treatment with cetuximab or panitumumab has been investigated in mainly retrospective cohort studies[19] [39] [40] [41] [42] [43] [44] [46]. Across these studies, the incidence of *BRAF *sequence variants ranged from 4.3% to 14.9% of patients with CRC. Two of these studies only reported data from patients who had previously tested negative for *KRAS *sequence variants[39] [43]; however, since patients tend not to have both *KRAS *and *BRAF *sequence variants[19] [40] [42] [46], only including patients who are *KRAS *negative should not greatly affect the proportion of patients with *BRAF *sequence variants. Seven studies reported the response to treatment with cetuximab or panitumumab in patients with the *BRAF* p.Val600Glu sequence variants and observed the following responses: 1 of 34 patients (excluding the patient with the p.Asp549Gly sequence variant)[46]; 0 of 13 patients[39]; 0 of 11 patients[41]; 0 of 9 patients[19]; 0 of 5 patients[43]; 1 of 4 patients[44]; and 1 of 2 patients[42]. Across these 7 studies, only 3 of 78 patients with a *BRAF* p.Val600Glu sequence variant responded to treatment with cetuximab or panitumumab. Caution should be used in interpreting these data as there is an overlap in patients included in the study of De Roock and colleagues and other European studies,[46] and all studies were retrospective in design.
- The response to treatment with anti-EGFR monoclonal antibodies in patients without *KRAS* variants or the *BRAF *p.Val600Glu sequence variant ranged from 27.6% to 46.4% in the 6 studies that reported this figure[19] [40] [41] [42] [43] [46], with 4 studies finding significant difference in response rates between patients with and without the *BRAF *p.Val600Glu sequence variant[19] [39] [41] [46]. The presence of the *BRAF* p.Val600Glu sequence variant was found to be associated with progression-free survival in 4 studies[19] [41] [43] [46], but no association was found in 2 studies that reported this endpoint[39] [40]. Similarly, the presence of the *BRAF *p.Val600Glu sequence variant was found to be associated with overall survival in all of the 5 studies[39] [40] [41] [43] [46] that reported this endpoint.
- Available data suggest that CRC patients with the *BRAF *p.Val600Glu sequence variant rarely respond to treatment with cetuximab or panitumumab. However, data are only available from retrospective studies that consisted mostly of cohorts of patients, rather than from randomized trials. Only 4.3% to 14.9% of patients with CRC were found to have the *BRAF *p.Val600Glu sequence variant. As a consequence, the number of patients with *BRAF *sequence variants in the CRC population who are negative for *KRAS *sequence variants is small. This is much lower than the percentage of *KRAS *sequence variants found in the CRC population at 40% to 45%[11]. In the studies included in this review, a total of only 78 patients with the *BRAF *p.Val600Glu sequence variant were available to assess the response to treatment (and there was some overlap in patients between studies). Available data suggest that testing for the *BRAF* sequence variant has promise in predicting which patients will not respond to treatment with EGFR monoclonal antibody inhibitors. However, the retrospective nature of the studies combined with the low number of patients included in *BRAF* p.Val600Glu sequence variant testing means that confirmation in larger prospective studies is needed before routine testing for the *BRAF* p.Val600Glu sequence variant can be recommended.

##  **Links**

- **Clinical Trials.gov:** **BRAF and colorectal cancer**
- **Online Mendelian Inheritance in Man:BRAF**
- **U.S Food and Drug Administration:Table of Valid Genomic Biomarkers in the Context of Approved Drug Labels**

Last updated: October 17, 2010

## **Acknowledgments**

The authors would like to acknowledge the contributions of the members of the Hayes Genetic Test Evaluation team, particularly Lisa Spock, Linnie Wieselquist and Charlotte Kuo-Benitez.

## **Funding information**

Funding for the Health Technology Assessment that informed this work was provided by Hayes, Incorporated. Funding to create this Knol was provided by the Centers for Disease Control and Prevention under Contract No. 200-2009-F-32675.This funding was provided through the Genetic Alliance.

## **Competing interests**

The authors are employees at Hayes, Inc., an independent health technology research and consulting company. None of the employees at this company has any financial or personal interest in any of the technologies reviewed by Hayes, Inc. No input on report content or conclusions is permitted by manufacturers. Although the CDC funded the work to produce this article, the content is based entirely on Hayes, Inc.'s own analysis and there was no input from the CDC.
